# The assistive wearable BrainPatch’s neurotechnology for an individual’s mental well-being

**DOI:** 10.1192/j.eurpsy.2026.11744

**Published:** 2026-07-31

**Authors:** N. Vysokov, D. Toleukhanov, Y. Gachshenko, S. Tukaiev

**Affiliations:** 1 BrainPatch, Cambridge, United Kingdom; 2 Educational Scientific Institute of High Technologies, Taras Shevchenko National University of Kyiv, Kyiv, Ukraine

## Abstract

**Introduction:**

Noninvasive neuromodulation techniques offer significant patient-friendly and cost advantages compared to traditional invasive methods. Effective control of autonomic activity through vagus nerve stimulation (VNS) may offer benefits for individuals coping with stress. The available evidence highlights several key aspects of these technologies, their applications, and their benefits for both patients and healthcare systems. Despite its demonstrated effectiveness, non-invasive vagus nerve stimulation has a set of weak points and shortcomings, unsolved technical problems.

**Objectives:**

We aimed to examine a BrainPatch’s neurotechnology of vagus nerve stimulation for minimizing the negative impact of everyday stress, improve mood and copy emotional burnout and for enhanced brain function.

**Methods:**

A total of 73 volunteers, 18-49 years old, were recruited for the study. The set of 4 VNS was performed at intervals of 3 days.

**Results:**

The BrainPatch Headphone Stimulators (https://www.brainpatch.ai/) are a combination of wireless headphones and wireless non-invasive transcutaneous auricular vagus nerve stimulators (micropolarization). The stimulation zone included the mastoid area where the auricular branch of the vagus nerve exits the cranium by passing through the tympanomastoid fissure between the mastoid process and the tympanic part of the temporal bone, and divides into two branches (first one joins the posterior auricular nerve, the other spreads to the skin of the auricle area of the ear and to the posterior part of the ear canal). The BrainPatch VNS device proposes a personalized approach to stimulating both the right and left vagus nerve. A BrainPatch application for iOS or Android operating systems is using for randomisation and for delivery of assigned protocols to the BrainPatch device as well as for collection of data on the execution of the protocol. The electrical stimulator (electrodes built into the headphones) consists of an analog-to-digital converter and a circuit that allows any signal to be delivered up to +/- 1.5 mA and up to ~ +/- 30 V to the electrodes. For the safety of stimulation, in addition to restrictions in the application, in the electronics itself there are 2 mA limiters in both directions. For stress-related and mental disorders treatment an application combines pleasant meditative classical music and a slow bi-polar wave (0.1-0.2 Hz, strength of current 0.25-1.5 mA, current frequency - 20-30 Hz, stimulation duration 5 min) of electrical stimulation, which produces a relaxing effect.

**Conclusions:**

The collected data suggest that the novel noninvasive device could benefit patients of all ages with mental and stress-related disorders.

**Disclosure of Interest:**

N. Vysokov Shareholder of: CEO, BrainPatch, D. Toleukhanov Shareholder of: CEO, BrainPatch, Y. Gachshenko Consultant of: BrainPatch Chief Technical Officer, S. Tukaiev Consultant of: BrainPatch Chief Neuroscientist

